# The Impact of Trait Mindfulness on Quality of Life in Patients With Bipolar Disorder: The Serial Mediating Role of Emotion Dysregulation and Depressive/Manic Symptoms

**DOI:** 10.62641/aep.2299

**Published:** 2026-10-15

**Authors:** Yangyang Xu

**Affiliations:** ^1^Xiamen Xianyue Hospital, Xianyue Hospital Affiliated With Xiamen Medical College, Fujian Psychiatric Center, Fujian Clinical Research Center for Mental Disorders, 361012 Xiamen, Fujian, China

**Keywords:** bipolar disorder, mindfulness, quality of life, emotion dysregulation, mood symptoms

## Abstract

**Background:**

To explore the impact of trait mindfulness on the quality of life (QoL) in patients with bipolar disorder (BD) and to examine the serial mediating roles of emotion dysregulation and depressive/manic symptoms.

**Methods:**

A cross-sectional study based on previous medical record data was conducted. Clinical records and scale data of 256 patients with BD who received outpatient or inpatient care at Xiamen Xianyue Hospital (Xiamen Mental Health Center) between June 2022 and June 2024 were extracted, all of whom carried a Diagnostic and Statistical Manual of Mental Disorders, Fifth Edition (DSM-5)-confirmed diagnosis. All scale assessments had been completed at the time of the index clinical visit, including the Five Facet Mindfulness Questionnaire (FFMQ), the Difficulties in Emotion Regulation Scale (DERS), the Self-Rating Depression Scale (SDS), the Bech-Rafaelsen Mania Scale (BRMS), and the Brief Quality of Life in Bipolar Disorder scale (Brief QoL.BD). Age, sex, bipolar subtype, illness duration, current mood state, and current pharmacological treatment were entered as covariates. Two separate serial mediation models (one with depressive symptoms and one with manic symptoms as the second-stage mediator) were tested using regression-based mediation analysis with bias-corrected bootstrapping (5000 resamples) under the PROCESS framework (Model 6).

**Results:**

Correlation analysis showed that trait mindfulness in patients with BD was significantly and positively correlated with QoL (r = 0.472, *p* < 0.001), and significantly and negatively correlated with emotion dysregulation (r = –0.523, *p* < 0.001), depressive symptoms (r = –0.457, *p* < 0.001), and manic symptoms (r = –0.406, *p* < 0.001). Serial mediation analysis revealed that the indirect effect of trait mindfulness on QoL through emotion dysregulation and depressive symptoms was 0.086 (95% CI [0.047, 0.132]), with a model explanatory power (adjusted R^2^) of 0.385. The indirect effect through emotion dysregulation and manic symptoms was 0.052 (95% CI [0.016, 0.091]), with a model explanatory power (adjusted R^2^) of 0.307.

**Conclusions:**

In this cross-sectional study based on previous medical record data, emotion dysregulation together with mood symptoms jointly served as a serial mediating chain linking trait mindfulness to QoL in patients with BD, and the depression-related chain conveyed a stronger mediating effect than the mania-related chain. Above findings suggest that a higher level of trait mindfulness may be associated with better QoL through enhanced emotion regulation and milder depressive/manic symptoms, with the depressive pathway being the most salient. The direction of these pathways still needs further verification by prospective designs.

## Introduction

Bipolar disorder (BD) is a chronic and recurrent psychiatric condition that imposes a heavy burden of disability. Beyond depressive and manic episodes, patients frequently display persistent functional impairment and diminished quality of life (QoL) even during inter-episode intervals [1]. In recent years, the prevailing clinical view has increasingly stressed that long-term treatment targets in BD should jointly address subjective QoL and the restoration of social functioning [2, 3]. Accumulating evidence indicates that, even when patients are relatively stable, their QoL remains well below that of the general population and is jointly influenced by sleep disturbances, cognitive load, and residual mood symptoms [4, 5]. Clarifying the key mechanisms—particularly protective factors and their pathways—that shape QoL in BD is therefore crucial for refining clinical intervention.

Trait mindfulness denotes an individual’s relatively stable disposition to attend to present-moment experience in daily life with open awareness and a non-judgmental stance [6]. It has recently been recognized as an important protective factor for emotion regulation and functional recovery in BD. Earlier work on euthymic BD has indicated that a lower level of trait mindfulness is coupled with poorer QoL and a greater tendency toward mood symptom recurrence [7]. Nevertheless, direct evidence on how trait mindfulness relates to QoL in BD is still limited, and most existing studies have examined only direct effects, leaving the underlying pathways largely unexplored.

Emotion dysregulation is widely considered a transdiagnostic mechanism of central importance in BD. A growing body of evidence shows that BD patients present broad difficulties across domains such as non-acceptance of emotional responses, poor impulse control, and limited emotional clarity, and that such regulatory abnormalities are consistently related to both the depressive and the manic dimensions of mood symptoms [8, 9]. Recent evidence suggests that dispositional mindfulness is associated with emotion regulation strategies and mood symptom dimensions in BD, and that maladaptive regulation strategies may mediate the relationship between mindfulness and depressive or anxiety symptoms [10]. In this context, people with higher trait mindfulness may relate to negative emotions with a more accepting and non-reactive attitude, thereby reducing emotion dysregulation and potentially contributing to better QoL. Although this mediating pathway has received empirical support, most of the existing work has relied on simple mediation models and has not yet teased apart how the depressive and manic symptom chains differ.

On the other hand, depressive and manic symptoms appear to shape QoL in BD through distinct mechanisms. Recent evidence from a large BD cohort suggests that residual depressive symptoms are associated with QoL and may represent an important target for improving functioning and QoL in BD [11]. Even though emotion dysregulation is associated with both symptom types, their mediating positions along the chain “trait mindfulness → emotion dysregulation → QoL” may diverge: emotion dysregulation could act on depressive and manic symptoms sequentially, which then shape QoL via two parallel serial paths. To date, however, few studies have simultaneously incorporated both symptom types into a single mediation framework to clarify their specific roles within the trait-mindfulness-to-QoL linkage.

Against this background, the present study adopts a cross-sectional study based on previous medical record data and a serial mediation framework, in which trait mindfulness (Five Facet Mindfulness Questionnaire (FFMQ)) serves as the predictor (X), QoL (12-item short form of the Brief Quality of Life in Bipolar Disorder scale (Brief QoL.BD-12)) as the outcome (Y), emotion dysregulation (Difficulties in Emotion Regulation Scale (DERS)) as the first-level mediator (M1), and depressive (Self-Rating Depression Scale (SDS)) and manic symptoms (Bech-Rafaelsen Mania Scale (BRMS)) as two second-level mediators (M2), respectively. We aim to examine two serial mediation pathways: trait mindfulness → emotion dysregulation → depressive/manic symptoms → QoL. Two hypotheses are proposed: (1) emotion dysregulation mediates the trait-mindfulness-QoL link; and (2) depressive and manic symptoms each form a separate serial chain downstream of emotion dysregulation—that is, mindfulness acts on QoL through emotion dysregulation and then, in turn, via depressive or manic symptoms. By distinguishing these two mediation pathways, the study seeks to provide more nuanced empirical evidence for mechanistic research on QoL in BD and to offer a theoretical basis for clinical interventions centered on mindfulness and emotion regulation.

## Materials and Methods

### Study Design

This study was designed as a cross-sectional study based on previous medical record data. Clinical records and psychological assessment data of patients with BD were extracted from the electronic medical record system and the affiliated psychological assessment database of Xiamen Xianyue Hospital (Xiamen Mental Health Center). The study covered patients attending Xiamen Xianyue Hospital (Xiamen Mental Health Center) from June 2022 to June 2024.

### Inclusion and Exclusion Criteria

Inclusion criteria: (1) aged 18 years or older; (2) medical records documenting a diagnosis of BD (type I or II) established according to the Diagnostic and Statistical Manual of Mental Disorders, Fifth Edition (DSM-5) [12] and confirmed by at least two attending-level (or higher) psychiatrists; (3) complete clinical documentation, with all required scale assessments on file.

Exclusion criteria: (1) comorbidity with schizophrenia spectrum disorders, schizoaffective disorder, or other severe psychiatric disorders; (2) severe neurological conditions, organic brain disorders, or major physical illnesses; and (3) dependence on or abuse of alcohol or other psychoactive substances.

### Participants

From these databases, 256 patients with BD who fulfilled the eligibility criteria were identified through a sequential records-screening process. A total of 270 potentially relevant records with a preliminary BD-related diagnosis were initially retrieved. During the eligibility verification process, 14 records were removed because they did not meet the predefined inclusion criteria, mainly due to incomplete scale records or insufficient documentation for DSM-5 diagnostic confirmation. Finally, 256 patients fulfilled all inclusion criteria and were included in the analysis. Among the 256 included patients, 158 (61.7%) were outpatients and 98 (38.3%) were inpatients at the time of assessment. As to the current mood state at the index visit, classification was determined according to psychiatrists’ clinical diagnosis based on DSM-5 criteria, complete medical history, mental status examination, and chart documentation: 132 patients (51.6%) were in a euthymic state, 78 (30.5%) in a depressive episode, and 46 (17.9%) in a manic or hypomanic episode. Sociodemographic data, clinical information, and psychological assessment data were then collated.

### Measures

#### General Information Questionnaire

The general information questionnaire was developed based on a literature review and clinical experience. Sociodemographic variables included sex, age, ethnicity, educational level, marital status, and employment status. Disease-related variables included history of psychiatric disorders, current diagnosis, duration of illness, family history, hospitalization status, and current pharmacological treatment.

#### FFMQ

Trait mindfulness was measured with the FFMQ originally developed by Baer *et al*. [6]. The instrument contains 39 items loading on five facets—observing, describing, acting with awareness, non-judging of inner experience, and non-reactivity to inner experience—each rated on a 5-point Likert-type scale. Total scores range from 39 to 195, with higher values denoting a stronger trait-level tendency toward mindfulness. In the present sample, Cronbach’s α of the total scale was 0.89, and Cronbach’s α of the five subscales ranged from 0.78 to 0.88.

#### DERS

Difficulties in emotion regulation were assessed with the DERS originally developed by Gratz and Roemer [13]. This questionnaire comprises 36 items grouped into six subscales: non-acceptance of emotional responses, difficulty engaging in goal-directed behavior, difficulty with impulse control, lack of emotional awareness, limited access to regulation strategies, and lack of emotional clarity. Each item is scored on a 5-point scale; summed scores range from 36 to 180, with higher values indicating more pronounced regulation difficulties. In the present sample, internal consistency of the full scale was satisfactory (Cronbach’s α = 0.91).

#### SDS

Depressive symptoms were quantified with the SDS introduced by Zung [14]. The scale consists of 20 items tapping affective, somatic, psychomotor, and psychological dimensions of depression, each scored on a 4-point Likert format. Raw scores span 20–80 and are converted to a standard score (raw × 1.25) falling between 25 and 100, with higher standard scores reflecting greater severity of depressive symptomatology. In the present sample, internal consistency was satisfactory (Cronbach’s α = 0.86).

#### BRMS

Manic symptoms were rated using the BRMS, originally described by Bech *et al*.[15]. This 11-item clinician-administered instrument uses a 0–4 scoring format for each item, so that total scores fall within 0–44; a higher total reflects a more severe manic state. In the present sample, internal consistency was acceptable (Cronbach’s α = 0.83). SDS and BRMS were used only as continuous measures of depressive and manic symptom severity in the correlation and mediation analyses, rather than as diagnostic tools or criteria for mood-state classification.

#### Brief Quality of Life in Bipolar Disorder (Brief QoL.BD)

QoL was evaluated using the Brief QoL.BD-12, devised by Michalak *et al*. [16]. The instrument samples 12 life domains—physical health, sleep, enjoyment, cognition, leisure, social relationships, self-esteem/motivation, finances, household activities, social acceptance, mobility, and self-identity—each scored on a 5-point Likert scale. Total scores range from 12 to 60, with higher scores denoting better perceived QoL. In the present sample, internal consistency was excellent (Cronbach’s α = 0.89).

### Data Collection and Processing

Two trained investigators independently reviewed outpatient and inpatient medical records collected at Xiamen Xianyue Hospital (Xiamen Mental Health Center) between June 2022 and June 2024. The extracted data covered: (1) sociodemographic characteristics; (2) disease-related information; and (3) psychological assessment data (scores on the FFMQ [6], DERS [13], SDS [14], BRMS [15], and Brief QoL.BD [16]). Every record was cross-checked before being entered into a unified database.

### Statistical Analysis

Data cleaning and statistical analyses were carried out using Python 3.11 (Python Software Foundation, Wilmington, DE, USA), together with the pandas, statsmodels, and pingouin libraries. Mediation analyses were implemented under the PROCESS macro framework (Model 6) re-coded in Python. As to specific procedures, the analyses included:

(1) Descriptive analysis: continuous variables were expressed as mean ± standard deviation (SD), and categorical variables were expressed as frequencies and percentages. For Table 1, QoL.BD scores were treated as continuous outcomes. Differences in QoL.BD scores across categorical grouping variables were examined using independent-samples *t*-tests for two-group variables and one-way analysis of variance (ANOVA) for variables with three or more groups. Welch’s correction was applied when the homogeneity of variance assumption was not met. Associations between continuous baseline variables, such as age and illness duration, and QoL.BD scores were assessed using Spearman correlation analysis. Normality of continuous variables was assessed using the Shapiro-Wilk test combined with visual inspection of Q-Q plots. The main continuous variables used in the Pearson correlation analysis, including FFMQ, DERS, SDS, BRMS, and Brief QoL.BD scores, showed approximately normal distributions; therefore, Pearson correlation analysis was considered appropriate for examining their bivariate linear associations.

**Table 1.** **Baseline characteristics and univariate analysis of QoL in included patients (n = 256)**.

Characteristic		Value (n = 256)	QoL.BD score	Statistic	*p* value
Age (years)		38.4 ± 13.7	—	r = –0.057	0.368
Sex, n (%)				*t* = –0.212	0.832
	Male	110 (43.0)	41.99 ± 8.59		
	Female	146 (57.0)	42.22 ± 8.57		
Ethnicity, n (%)				*t* = 1.091	0.289
	Han	238 (93.0)	42.28 ± 8.55		
	Other	18 (7.0)	39.97 ± 8.67		
Educational level, n (%)				F = 0.654	0.521
	Junior high school or below	47 (18.4)	43.19 ± 7.96		
	Senior high school/vocational	92 (35.9)	41.45 ± 9.02		
	College or above	117 (45.7)	42.21 ± 8.44		
Marital status, n (%)				*t* = –1.641	0.102
	Single/divorced/widowed	118 (46.1)	41.17 ± 8.72		
	Married	138 (53.9)	42.93 ± 8.36		
Employment status, n (%)				*t* = 0.407	0.684
	Employed/student	146 (57.0)	42.31 ± 8.64		
	Unemployed/retired/on sick leave	110 (43.0)	41.87 ± 8.49		
Setting at index assessment, n (%)				*t* = –0.648	0.518
	Outpatient	158 (61.7)	41.85 ± 8.82		
	Inpatient	98 (38.3)	42.55 ± 8.14		
Bipolar subtype, n (%)				*t* = –1.462	0.145
	Bipolar I disorder	167 (65.2)	41.58 ± 9.04		
	Bipolar II disorder	89 (34.8)	43.13 ± 7.52		
Illness duration (years)		15.6 ± 7.1	—	r = –0.104	0.097
Current mood state, n (%)				F = 34.948	<0.001
	Euthymic	132 (51.6)	45.79 ± 7.52		
	Depressive episode	78 (30.5)	39.53 ± 7.55		
	Manic/hypomanic episode	46 (17.9)	35.97 ± 7.93		
History of psychiatric disorders prior to BD diagnosis, n (%)				*t* = –0.793	0.429
	Yes	63 (24.6)	41.44 ± 7.43		
	No	193 (75.4)	42.34 ± 8.90		
Family history of psychiatric disorders, n (%)				*t* = 0.128	0.898
	Yes	71 (27.7)	42.23 ± 8.25		
	No	185 (72.3)	42.08 ± 8.70		
Prior psychiatric hospitalization, n (%)				*t* = 0.378	0.706
	Yes	112 (43.8)	42.35 ± 8.70		
	No	144 (56.2)	41.94 ± 8.48		
Current pharmacological treatment, n (%)				F = 47.253	<0.001
	Mood stabilizer monotherapy	76 (29.7)	49.49 ± 6.03		
	Mood stabilizer + antipsychotic	104 (40.6)	40.51 ± 7.09		
	Mood stabilizer + antidepressant	48 (18.8)	35.43 ± 7.97		
	Others/combinations	28 (10.9)	39.59 ± 6.28		

Continuous variables are presented as mean ± standard deviation (SD), and categorical variables as n (%). QoL.BD scores are presented as mean ± SD. Differences in QoL.BD scores across categorical variables were analyzed using independent-samples *t*-tests or one-way ANOVA, as appropriate; Welch’s correction was applied when the homogeneity of variance assumption was not met. Associations between continuous baseline variables and QoL.BD scores were assessed using Spearman correlation analysis. BD, bipolar disorder; QoL.BD, Quality of Life in Bipolar Disorder.

(2) As the main scale scores were approximately normally distributed, Pearson correlation analysis was applied to examine the linear relationships among trait mindfulness, emotion dysregulation, depressive symptoms, manic symptoms, and QoL. As Pearson correlation only reflects unadjusted bivariate linear relationships and does not control for confounders, it was used solely for preliminary description and was not relied upon as the primary method for mediation inference;

(3) Regression-based serial mediation analyses were performed under the PROCESS Model 6 framework. Two separate serial mediation models were specified rather than a single parallel-mediator model: in Model A, trait mindfulness (X) → emotion dysregulation (M1) → depressive symptoms (M2) → QoL (Y); in Model B, trait mindfulness (X) → emotion dysregulation (M1) → manic symptoms (M2) → QoL (Y). Above two models were estimated independently to avoid conceptual confusion between parallel and serial mediation, and the wording “parallel and sequential pathways” used in the earlier version has been corrected accordingly. Age, sex, bipolar subtype (BD I vs BD II), illness duration, current mood state (euthymic/depressive episode/manic-hypomanic episode), and current pharmacological treatment (mood stabilizer only/mood stabilizer plus antipsychotic/mood stabilizer plus antidepressant/others) were entered as covariates in all path equations. Before model estimation, regression assumptions were systematically checked: normality of residuals was assessed using the Shapiro-Wilk test and Q-Q plots; multicollinearity was screened using the variance inflation factor (VIF), with VIF < 5 considered acceptable; and heteroscedasticity was examined using the Breusch-Pagan test. Standardized coefficients (β) were reported consistently for all path estimates. Indirect effects were estimated using bias-corrected bootstrapping with 5000 resamples to derive 95% confidence intervals (95% CI); an indirect effect was considered statistically significant when the 95% CI did not include zero. Above all, model performance was reported using adjusted R^2^ together with standardized residual diagnostics rather than R^2^ alone, since R^2^ in isolation is inadequate for path/mediation models. Two-sided *p* < 0.05 was taken as the threshold of statistical significance.

## Results

### Common Method Bias Test

Potential common method bias was examined using Harman’s single-factor test. The first unrotated factor accounted for 27.631% of the total variance, which was below the commonly used 50% criterion for this test [17, 18], suggesting that no dominant single factor was present. As to the limitation of Harman’s single-factor test, it has been recognized that this approach is relatively crude and may not fully detect inflation introduced by self-report measurement. Therefore, above result should be interpreted as a preliminary screen only, and the potential inflation of associations due to common method variance is further discussed as a limitation in section “Discussion”. More robust approaches, such as the confirmatory factor analysis (CFA) marker variable approach, still need further verification in subsequent multi-source datasets.

### Sample Characteristics

Of the 270 potentially relevant records initially screened, 14 records did not meet the eligibility requirements during data verification, and 256 valid cases were finally included, with an effective inclusion rate of 94.8%. The sample comprised 110 male (43.0%) and 146 female (57.0%) patients, aged between 20 and 68 years (mean = 38.4 ± 13.7 years). As to the clinical profile, 167 patients (65.2%) had bipolar I disorder and 89 (34.8%) had bipolar II disorder, with a mean illness duration of 15.6 ± 7.1 years. Detailed baseline demographic and clinical characteristics—including ethnicity, educational level, marital status, employment status, history of psychiatric disorders, family history of psychiatric disorders, prior hospitalization history, current mood state, and current pharmacological treatment—are summarized in Table 1. Univariate analysis showed that current mood state and current pharmacological treatment were significantly associated with QoL.BD scores (both *p* < 0.001), whereas age, sex, ethnicity, educational level, marital status, employment status, assessment setting, bipolar subtype, illness duration, history of psychiatric disorders prior to BD diagnosis, family history of psychiatric disorders, and prior psychiatric hospitalization were not significantly associated with QoL.BD scores (all *p* > 0.05).

### Descriptive Statistics and Correlation Analysis

The means, SDs, and bivariate Pearson correlation coefficients of all variables are presented in Table 2. Trait mindfulness was significantly positively correlated with QoL (r = 0.472, *p* < 0.001), and significantly negatively correlated with difficulties in emotion regulation (r = –0.523, *p* < 0.001), depressive symptoms (r = –0.457, *p* < 0.001), and manic symptoms (r = –0.406, *p* < 0.001). Above correlations are unadjusted and were used solely for preliminary description; covariate-adjusted estimates are reported in section “Chain Mediation Analysis”.

**Table 2.** **Means, SDs, and correlation matrix of all variables (n = 256)**.

Variable	Mean	SD	1	2	3	4	5
1 Trait mindfulness	123.60	17.73	1				
2 Difficulties in emotion regulation	99.05	12.41	–0.523***	1			
3 Depressive symptoms	57.09	12.65	–0.457***	0.588***	1		
4 Manic symptoms	12.89	5.34	–0.406***	0.469***	0.418***	1	
5 QoL	42.12	8.56	0.472***	–0.517***	–0.504***	–0.485***	1

QoL, quality of life; SD, standard deviation. Values on the diagonal are 1.000 (self-correlation). ***p < 0.001.

Difficulties in emotion regulation were significantly positively correlated with depressive symptoms (r = 0.588, *p* < 0.001) and manic symptoms (r = 0.469, *p* < 0.001). Both depressive and manic symptoms were significantly negatively correlated with QoL (r = –0.504 and –0.485, respectively; *p* < 0.001).

### Chain Mediation Analysis

#### Model A: Depression Pathway

Model A was adjusted for age, sex, bipolar subtype, illness duration, current mood state, and current pharmacological treatment. Assumption checks indicated that residuals were approximately normal (Shapiro-Wilk W = 0.987, *p* = 0.073), all VIF values were below 2.6, and the Breusch-Pagan test did not reveal severe heteroscedasticity (*p* = 0.118), supporting the validity of the regression-based mediation framework. After covariate adjustment, trait mindfulness still exerted a significant total effect on QoL (c = 0.317, SE = 0.041, *p* < 0.001). After including the two mediators, the direct effect remained significant (c^′^ = 0.119, SE = 0.045, *p* = 0.008), indicating a partial mediation effect. Path analysis (Fig. 1) showed that trait mindfulness significantly negatively predicted difficulties in emotion regulation (β = –0.468, *p* < 0.001) and depressive symptoms (β = –0.053, *p* = 0.037). Difficulties in emotion regulation significantly positively predicted depressive symptoms (β = 0.421, *p* < 0.001) and negatively predicted QoL (β = –0.190, *p* = 0.002). Depressive symptoms were also significantly negatively related to QoL (β = –0.436, *p* < 0.001). Bias-corrected bootstrap analysis with 5000 resamples (see Table 3) indicated that all three indirect pathways were significant: (1) Trait mindfulness → difficulties in emotion regulation → QoL (indirect effect = 0.089, 95% CI [0.049, 0.132], accounting for 28.08% of the total effect); (2) Trait mindfulness → depressive symptoms → QoL (indirect effect = 0.023, 95% CI [0.005, 0.044], 7.26% of the total effect); (3) Trait mindfulness → difficulties in emotion regulation → depressive symptoms → QoL (chain indirect effect = 0.086, 95% CI [0.047, 0.132], 27.13% of the total effect). The total indirect effect was 0.198 (95% CI [0.132, 0.267], 62.47% of the total effect), and the model explained 38.5% of the variance in QoL (adjusted R^2^ = 0.385).

**Fig. 1.**
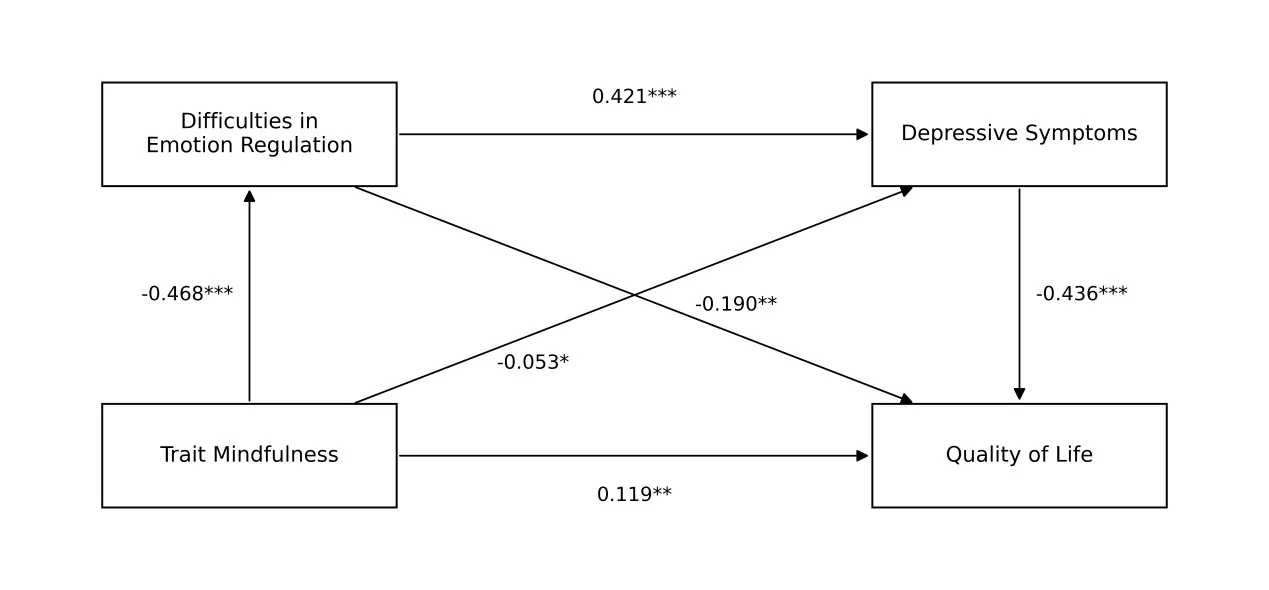
**Serial mediation model for the depression pathway (Model A)**. Path coefficients are standardized β values, adjusted for age, sex, bipolar subtype, illness duration, current mood state, and current pharmacological treatment. ***p < 0.001, **p < 0.01, *p < 0.05.

**Table 3.** **Bootstrap mediation effects for Model A—depression pathway (n = 256)**.

Path and effect	Effect	SE	95% CI	Proportion (%)
Total effect (c)	0.317	0.041	[0.237, 0.397]	100.00%
Direct effect (c′)	0.119	0.045	[0.031, 0.207]	37.53%
Total indirect effect	0.198	0.034	[0.132, 0.267]	62.47%
X→M1→Y	0.089	0.021	[0.049, 0.132]	28.08%
X→M2 (Depression)→Y	0.023	0.01	[0.005, 0.044]	7.26%
X→M1→M2 (Depression)→Y	0.086	0.021	[0.047, 0.132]	27.13%

Indirect effects were estimated using bias-corrected bootstrapping with 5000 resamples. X = Trait mindfulness (FFMQ); M1 = Difficulties in emotion regulation (DERS); M2 = Depressive symptoms (SDS); Y = QoL (Brief QoL.BD). CI, confidence interval; SE, standard error. Models were adjusted for age, sex, bipolar subtype, illness duration, current mood state, and current pharmacological treatment.

#### Model B: Mania Pathway

In the mania pathway model, regression assumptions were likewise satisfied (Shapiro-Wilk W = 0.984, *p* = 0.052; VIF < 2.4; Breusch-Pagan *p* = 0.156) after adjustment for the same covariate set. Path analysis (see Fig. 2) showed that trait mindfulness significantly negatively predicted difficulties in emotion regulation (β = –0.468, *p* < 0.001) and manic symptoms (β = –0.043, *p* = 0.048). Difficulties in emotion regulation significantly positively predicted manic symptoms (β = 0.338, *p* < 0.001) and negatively predicted QoL (β = –0.196, *p* = 0.001). Manic symptoms were significantly negatively related to QoL (β = –0.328, *p* = 0.002). The direct effect remained significant (c^′^ = 0.159, SE = 0.046, *p* < 0.001). Bias-corrected bootstrap results (see Table 4) revealed that all indirect effects were significant: (1) Trait mindfulness → difficulties in emotion regulation → QoL (indirect effect = 0.092, 95% CI [0.050, 0.137], 29.02% of the total effect); (2) Trait mindfulness → manic symptoms → QoL (indirect effect = 0.014, 95% CI [0.002, 0.030], 4.42% of the total effect); (3) Trait mindfulness → difficulties in emotion regulation → manic symptoms → QoL (chain indirect effect = 0.052, 95% CI [0.016, 0.091], 16.43% of the total effect). The total indirect effect was 0.158 (95% CI [0.101, 0.219], 49.87% of the total effect), and the model explained 30.7% of the variance in QoL (adjusted R^2^ = 0.307).

**Table 4.** **Bootstrap mediation effects for Model B—mania pathway (n = 256)**.

Path and effect	Effect	SE	95% CI	Proportion (%)
Total effect (c)	0.317	0.041	[0.237, 0.397]	100.00%
Direct effect (c′)	0.159	0.046	[0.069, 0.249]	50.13%
Total indirect effect	0.158	0.03	[0.101, 0.219]	49.87%
X→M1→Y	0.092	0.022	[0.050, 0.137]	29.02%
X→M2 (Mania)→Y	0.014	0.007	[0.002, 0.030]	4.42%
X→M1→M2 (Mania)→Y	0.052	0.018	[0.016, 0.091]	16.43%

Indirect effects were estimated using bias-corrected bootstrapping with 5000 resamples. X = Trait mindfulness (FFMQ); M1 = Difficulties in emotion regulation (DERS); M2 = Manic symptoms (BRMS); Y = QoL (Brief QoL.BD). CI, confidence interval; SE, standard error. Models were adjusted for age, sex, bipolar subtype, illness duration, current mood state, and current pharmacological treatment.

**Fig. 2.**
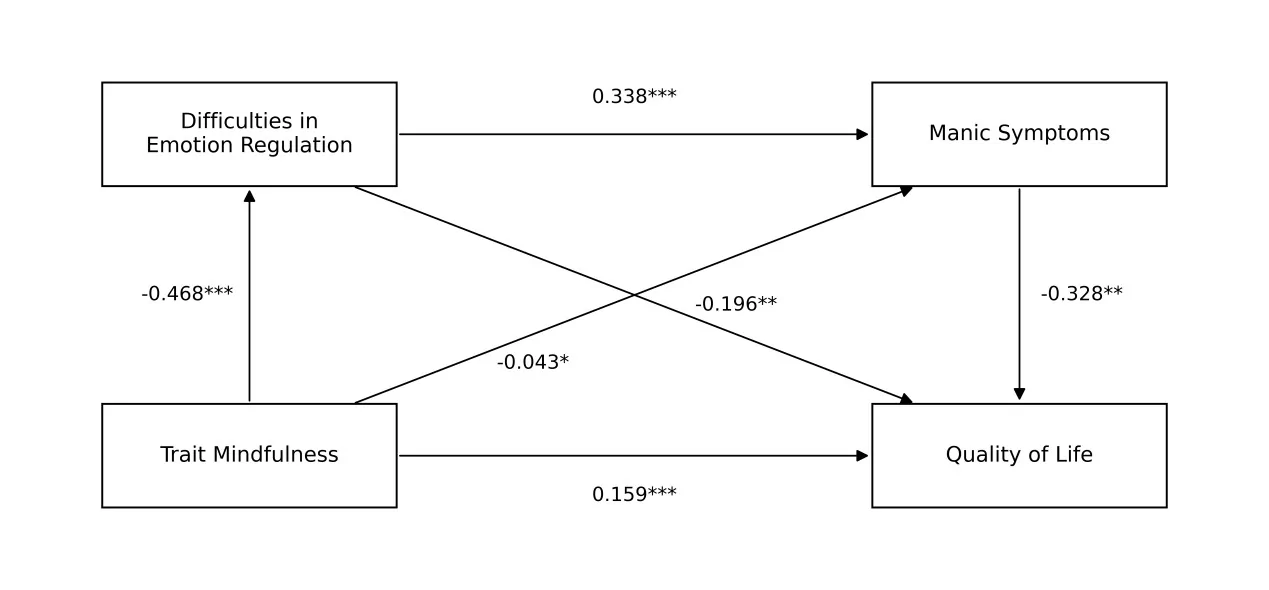
**Serial mediation model for the mania pathway (Model B)**. Path coefficients are standardized β values, adjusted for age, sex, bipolar subtype, illness duration, current mood state, and current pharmacological treatment. ***p < 0.001, **p < 0.01, *p < 0.05.

## Discussion

This study examined the relationship between trait mindfulness and QoL in patients with BD and tested the serial mediating roles of difficulties in emotion regulation and depressive/manic symptoms within a cross-sectional study based on previous medical record data. The findings supported the hypothesized associative model: trait mindfulness was not only directly and positively associated with QoL, but was also linked to it indirectly through two separate serial pathways involving difficulties in emotion regulation and emotional symptoms. The mediating effect of the depression-related serial chain was stronger than that of the mania-related chain, suggesting that, in this sample, the favorable association between trait mindfulness and QoL in BD patients was more closely related to depression-related emotion regulation difficulties. As above analysis showed, the present results provide preliminary empirical evidence for understanding the mechanisms potentially underlying trait mindfulness and offer implications for clinical intervention, while the directionality of these pathways still needs further verification.

Above analysis suggested that trait mindfulness was moderately positively correlated with QoL in BD patients, and its direct association remained significant after covariate adjustment and inclusion of the mediators. This finding is consistent with recent evidence suggesting that mindful awareness is associated with residual mood symptoms and functional status in patients with BD [19]. Functional recovery and QoL remain important treatment-related outcomes in BD [20]. One possible explanation is that individuals with a higher level of trait mindfulness may be more capable of monitoring present-moment experiences with acceptance, which can reduce affective reactivity and negative appraisal of illness-related limitations and may thereby be associated with better perceived QoL [7, 21]. Components of mindfulness, such as acting with awareness and non-reactivity, may be relevant to patients’ perceived QoL in BD. This interpretation is consistent with evidence linking dispositional mindfulness to life quality in BD and with the QoL.BD framework, which includes functioning-related and self-related quality-of-life domains [16, 22, 23]. From the angle of clinical rehabilitation assessment, trait mindfulness may therefore be regarded as a positive psychological resource that is statistically associated with—rather than causally independent of—symptom improvement; this associative interpretation is more consistent with the observational nature of the present design.

Difficulties in emotion regulation were found to significantly mediate the relationship between trait mindfulness and QoL, and this pathway was consistent across both the depression and mania models. This finding supports the notion that emotion regulation difficulties represent a transdiagnostic mechanism in BD. Recent intervention evidence in BD suggests that mindfulness-based psychoeducation can improve mindfulness and emotion regulation strategies in patients with BD [24]. In addition, prior work has indicated that emotion regulation difficulties or emotional regulation strategies may mediate the associations between trait mindfulness, mood symptoms, and QoL in BD [7, 10]. This pathway is closely linked to mindfulness facets such as nonjudgment and emotional clarity. Individuals with higher mindfulness are better able to identify and tolerate negative emotions without being overwhelmed by them. In both serial mediation models, the simple indirect pathway (trait mindfulness → difficulties in emotion regulation → QoL) accounted for the largest proportion of the total indirect effect, indicating that emotion regulation difficulties may partially explain the association between mindfulness and QoL in the present sample. This suggests that improving emotion regulation capacity may represent a direct and efficient intervention target for enhancing QoL in BD patients.

A key contribution of this study lies in simultaneously examining depressive and manic symptoms as secondary mediators within a serial mediation framework and comparing their relative effects. Although both serial pathways were significant, the depressive pathway demonstrated a stronger effect size and higher explanatory power than the manic pathway. This difference has important theoretical and clinical implications. QoL in BD patients appears to be closely related to depressive symptoms, which is consistent with recent evidence showing that residual or subsyndromal depressive symptoms are associated with impaired functioning and reduced QoL in BD [25, 26]. Furthermore, the present study reveals that trait mindfulness more effectively alleviates depressive symptoms through improvements in emotion regulation, thereby indirectly enhancing QoL. In contrast, the impact of manic symptoms on QoL is more complex: mild manic states may be associated with increased subjective well-being, whereas severe mania leads to substantial functional impairment. In the present relatively stable BD sample, manic symptoms remained negatively associated with QoL, but their mediating role was weaker. This may reflect the lower and residual nature of manic symptoms during non-acute phases; recent evidence suggests that manic residual symptoms are less frequent than depressive residual symptoms but should not be ignored [27, 28]. From a mechanistic perspective, the association between emotion regulation difficulties and depressive symptoms is stronger than that with manic symptoms, which may underlie the greater effect of the depressive pathway. From a mechanistic perspective, emotion regulation difficulties are closely associated with both depressive and manic symptom dimensions in BD, although the strength and pattern of these associations may vary across mood states and regulation strategies [29]. Trait mindfulness may exert a stronger buffering effect on the former, as mindfulness training emphasizes awareness and acceptance, which directly counteract experiential avoidance and cognitive fusion associated with depression [30, 31].

Above findings carry potentially meaningful implications for psychosocial interventions in BD, although such implications should be drawn cautiously given that the design was a cross-sectional study based on previous medical record data. Strengthening trait mindfulness may be considered as one candidate target in rehabilitation. Mindfulness-based cognitive therapy has previously been reported to be beneficial for emotional symptoms and functional recovery in BD [32], and acceptance and commitment therapy has also been explored as a psychological intervention for patients with BD [33]. The present associative findings offer additional mechanism-oriented support for interventions targeting mindfulness, acceptance, and emotion regulation. Interventions may prioritize the development of emotion regulation skills, particularly emotional acceptance, impulse control, and access to adaptive strategies [34], as above factors appeared to be the most prominent statistical links between mindfulness and symptom relief in this sample. Given the stronger depression-related serial chain, greater clinical attention should be paid to the early identification and management of depressive symptoms, even during relatively stable phases. For patients with prominent manic symptoms, mindfulness-based interventions may be enhanced by incorporating modules targeting impulse control and early emotional warning signals.

Several limitations should be acknowledged. First, this was a cross-sectional study based on previous medical record data, and all variables were assessed at a single time point; therefore, the temporal order and causal direction among trait mindfulness, emotion dysregulation, mood symptoms, and QoL could not be determined. Second, although several demographic and clinical covariates were adjusted for, residual confounding from factors such as comorbid anxiety, sleep quality, psychotherapy exposure, and medication dosage could not be fully excluded. Third, most variables were measured using self-report scales, which may introduce common method bias despite the preliminary screening result. Fourth, cognitive factors and dimensional features of manic symptoms were not assessed, which may limit the explanatory completeness of the mediation model. Finally, the sample was drawn from a single center with a moderate sample size, and the generalizability of the findings needs to be verified in larger, multi-center, longitudinal studies.

Based on the above analyses, the present study constructed two separate serial mediation models in BD patients and suggested that trait mindfulness was statistically related to QoL through difficulties in emotion regulation and emotional symptoms (depressive or manic). Difficulties in emotion regulation emerged as a core mediating mechanism, and the depression-related chain showed a significantly stronger mediating effect than the mania-related chain. Future research directions—such as prospective longitudinal verification, randomized controlled trials of mindfulness-based interventions, the inclusion of cognitive mediators (attention, working memory, executive function), the dimensional decomposition of manic symptoms (euphoria vs irritability/dysphoria), and the introduction of neurobiological markers and ecological momentary assessment—are discussed above and represent promising directions for clarifying the dynamic mechanisms by which trait mindfulness safeguards QoL in BD.

## Conclusions

In the present cross-sectional study based on previous medical record data involving 256 patients with BD, trait mindfulness was positively associated with QoL, and this association was conveyed serially through emotion dysregulation together with depressive or manic symptoms. The depression-related serial chain carried a larger mediating effect than the mania-related chain. Above findings, while restricted to associative inference, suggest that interventions targeting trait mindfulness and emotion regulation—particularly addressing depressive symptoms—may be promising candidates for improving QoL in BD and still need further verification in prospective, multi-center, controlled studies.

## Availability of Data and Materials

The datasets used and/or analysed during the current study are available from the corresponding author on reasonable request.
